# Development and validation of a nomogram for predicting overall survival in patients with early-onset endometrial cancer

**DOI:** 10.1186/s12885-023-11682-9

**Published:** 2023-12-14

**Authors:** Meng Zhang, Ruiping Li, Jiaxi Zhang, Yunyun Wang, Yunlu Wang, Yuzhen Guo

**Affiliations:** Department of Gynecology, Second Hospital of Lanzhou University, No.82 Cui Ying Gate, Cheng guan District, Lanzhou, Gansu 730030 China

**Keywords:** Early-onset endometrial cancer, Nomogram, Overall survival, Surveillance, epidemiology, and end results (SEER) database

## Abstract

**Background:**

This study aimed to investigate the differences in the clinicopathological characteristics of younger and older patients with endometrial cancer (EC) and develop a nomogram to assess the prognosis of early onset EC in terms of overall survival.

**Methods:**

Patients diagnosed with EC from the Surveillance, Epidemiology, and End Results (SEER) database between 2004 and 2015 were selected. Clinicopathological characteristics were compared between younger and older patients, and survival analysis was performed for both groups. Prognostic factors affecting overall survival in young patients with EC were identified using Cox regression. A nomogram was created and internal validation was performed using the consistency index, decision curve analysis, receiver operating characteristic curves, and calibration curves. External validation used data from 70 patients with early onset EC. Finally, Kaplan-Meier curves were plotted to compare survival outcomes across the risk subgroups.

**Results:**

A total of 1042 young patients and 12,991 older patients were included in this study. Younger patients were divided into training (732) and validation (310) cohorts in a 7:3 ratio. Cox regression analysis identified age, tumorsize, grade, FIGO stage(International Federation of Gynecology and Obstetrics) and surgery as independent risk factors for overall survival, and a nomogram was constructed based on these factors. Internal and external validations demonstrated the good predictive power of the nomogram. In particular, the C-index for the overall survival nomogram was 0.832 [95% confidence interval (0.797–0.844)] in the training cohort and 0.839 (0.810–0.868) in the internal validation cohort. The differences in the Kaplan-Meier curves between the different risk subgroups were statistically significant.

**Conclusions:**

In this study, a nomogram for predicting overall survival of patients with early onset endometrial cancer based on the SEER database was developed to help assess the prognosis of patients and guide clinical treatment.

**Supplementary Information:**

The online version contains supplementary material available at 10.1186/s12885-023-11682-9.

## Introduction

Endometrial cancer (EC) is the sixth most common cancer affecting women worldwide. In recent years, EC has become the most common gynecological malignancy in developed countries [1], and its incidence is increasing in young women [2]. Meanwhile, the incidence of EC and associated mortality are on the rise [3]. It is estimated that there were 66,570 new cases and 12,940 deaths from this disease in the United States in 2021 [4]. Although most women diagnosed with EC have already gone through menopause, premenopausal women younger than 45 account for approximately 10% of reported cases of EC [5].

Factors associated with an increased risk of EC include age, obesity, family history, and tumor stage [6]. Previous studies have suggested age as an important factor associated with overall survival, the importance of age in assessing the prognosis of patients with EC remains controversial [7]. It has been suggested that patients with EC under 45 years of age tend to have a lower incidence of advanced disease, a higher degree of tumor differentiation, and better prognosis [8]. Early diagnosis of EC in younger patients is suggested to be lower and can easily be misdiagnosed as abnormal uterine bleeding, whereas older patients are more likely to be diagnosed at an early stage [8]. Nelson et al. [9] found no association between increasing age and the prevalence of endometrial hyperplasia, which was more common in women aged < 40 years than in those aged 40–50 years. Clinically, patients younger than 45 years were defined as having early onset EC. The FIGO staging system proposed by the International Federation of Obstetrics and Gynecology has been widely used to predict the prognosis of EC [10]. However, the FIGO staging system remains limited and does not accurately predict the prognosis. Further comprehensive analysis of the prognostic factors associated with patients with early onset EC is necessary to establish individualized treatment plans.

In this study, differences in clinicopathological characteristics affecting the prognosis of younger and older patients were explored using data from the Surveillance, Epidemiology, and End Results (SEER) database. Variables associated with the prognosis of young patients were further analyzed. A nomogram was constructed using the relevant variables to predict the prognosis of patients with early onset EC at 3-, 5-, 8-, and 10-year overall survival and to guide clinical treatment.

## Materials and methods

### Training and internal validation cohorts

This study used the SEER database (http://seer.cancer.gov/seerstat/), which covers 30% of the population of the United States. SEER has collected information on incidence rate, morbidity, mortality and other evidence-based drugs of cancer patients in some states and counties in the United States for decades, providing valuable information about cancer diseases for most clinical medical personnel [11, 12]. This study used data from 1975 to 2020. Data were extracted, downloaded, and analyzed using SEER*Stat 8.4.1.2. (accunt ID: 18,893,816,203@163.com). Primary sites, C54.1-9 and C55.917; site and morphology, 8380/3 (based on the International Classification of Tumor Diseases for Oncology (ICD-O), Third Edition); histology, 8000–9930 (adenomas and adenocarcinomas); FIGO stage I,II,III and IV; and therapy. This study was approved by the Ethics Committee of the Second Hospital of Lanzhou University (Ethics Approval No.2022 A-336) and strictly complied with the Declaration of Helsinki. The Ethics Committee of the Second Hospital of Lanzhou University waived the requirement for informed consent.

**Fig. 1:**
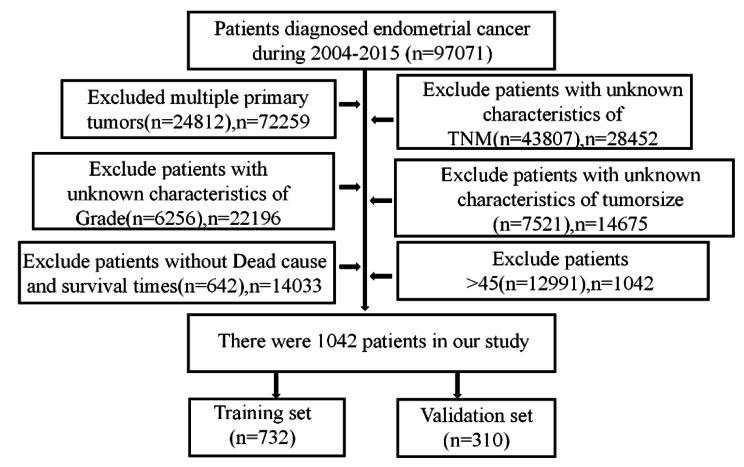
Flowchart for selection of research subjects

Patients diagnosed with early onset EC between 2004 and 2015 were included in this study. The exclusion criteria were as follows: (I) age > 45 years, (II) multiple primary tumors, (III) missing disease-related information (FIGO stage, grade and tumorsize), (IV) unclear cause of death, and (VI) incomplete follow-up time. The flowchart is shown in Fig. 1. In total, 1042 young patients and 12,991 older patients were selected from the SEER database. Younger patients were randomly (7:3) divided into training (n = 732) and validation (n = 310) cohorts. There were no significant differences in the variables between the two groups (all *p*-values < 0.05), as detailed in Table 1.

The variables included age at diagnosis, race, marital status, histological type, tumorsize, grade, FIGO stage, radiotherapy, chemotherapy, and surgery. The follow-up variables included survival status, survival time, and cause of death. The primary endpoint was overall survival, which refers to the time from diagnosis to death from any cause.Age (≤ 33 and 34–45 years) and tumorsize(≤ 4 and > 4 cm) were determined using x-tile software to obtain the optimal cutoff values. FIGO stage use 2009 version.

### External validation cohort

The clinical data of 70 patients who were treated at the Department of Gynecology of the Second Hospital of Lanzhou University and pathologically diagnosed with EC between January 2011 and August 2021 were retrospectively analyzed. The inclusion criteria were as follows: (I) age < 45 years; (II) primary tumor; (III) confirmed postoperative pathological diagnosis of EC; and (V) complete clinical and postoperative follow-up data. The follow-up was conducted on August 30, 2021.

### Data analysis

We first compared the basic clinical information of elderly EC patients and young EC patients, and then obtained factors that affect the prognosis of young EC patients based on Cox univariate regression. *P* < 0.05 was included in Cox multivariate regression for further analysis. Finally, a predictive model related to overall survival rate was constructed.The model predictive ability was evaluated using the concordance index, area under the receiver operating characteristic curve, and decision curve analysis. Calibration curves were plotted to assess the agreement between the nomogram and actual model. According to the total scores of the validation and training queues, patients are divided into low-risk and high-risk groups, respectively. Kaplan-Meier curves were used to compare overall survival between the two groups. All statistical analyses were performed using SPSS (version 26.0; SPSS Inc., Chicago, IL, USA) and R (version 4.3.1; R Foundation for Statistical Computing, Vienna, Austria).

## Results

**Fig. 2:**
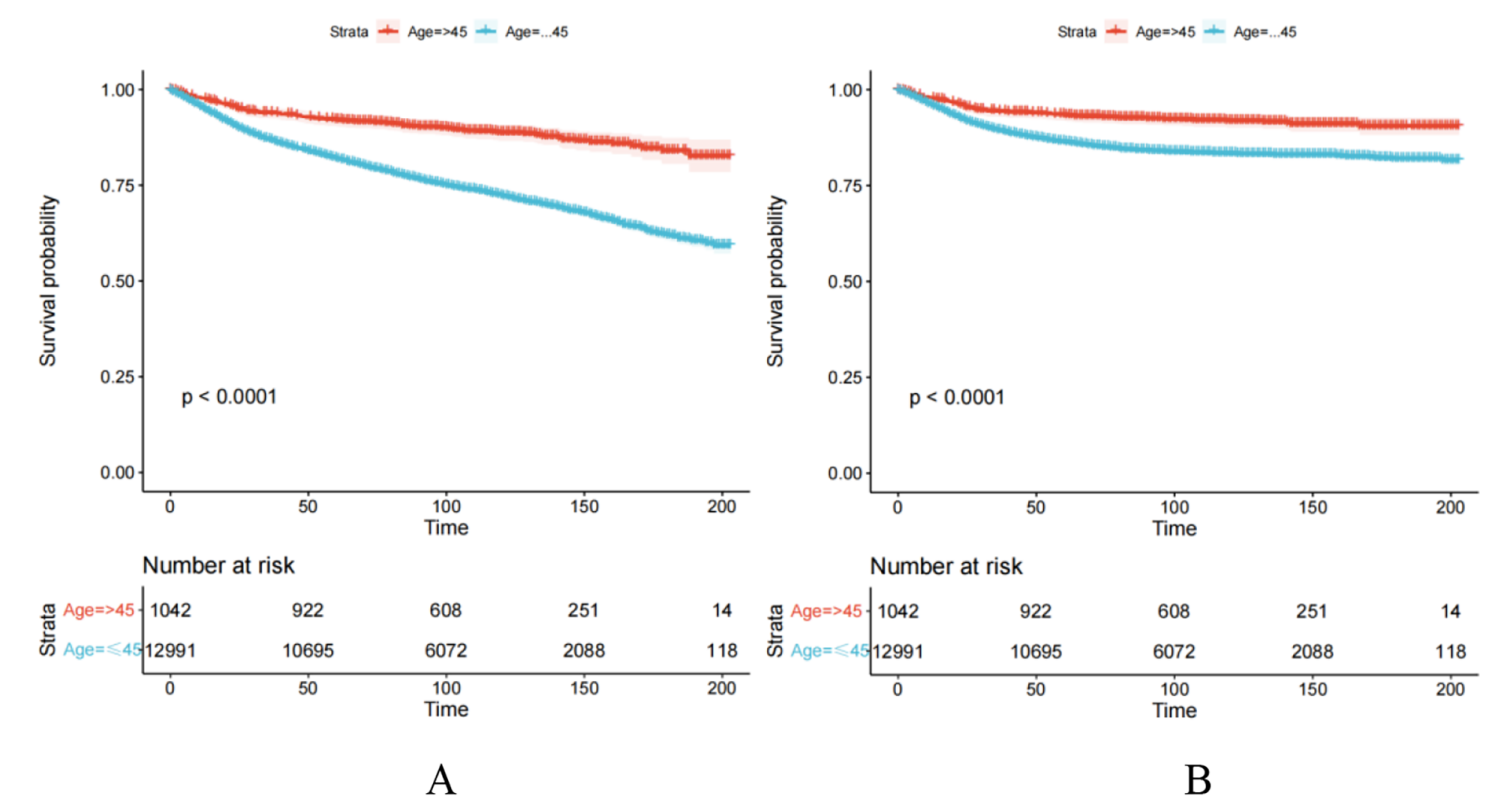
The overall survival (**A**: OS) and cancer specific survival (**B**: CSS) of younger and older patients

We included 1042 young patients and 12,991 older patients with EC from the SEER database. In terms of race, both groups had a high proportion of white patients, 61.0% and 78.6%, respectively. The histological type of adenocarcinoma accounted for 83.7% of the younger patients. Regarding the histological grades, a higher proportion of younger patients were in grade I (58.7%), and a higher number of older patients were in grade II or above (55.7%).Many patients were located in FIGO I. The numbers of young and older patients receiving adjuvant treatment were low (Table 1). Survival analysis showed that overall survival and cancer-specific survival were higher in younger patients than in older patients (*P*-value < 0.001) (Fig. 2).

**Table 1:** Demographic and clinicopathological characteristics among young and older EC patients

Variables	age ≤ 45 years old (n = 1042)		age > 45 years old (n = 12,991)		*P* value
	N	%	N	%	
Marital status					
Married	507	48.7	6913	53.2	0.004
Other	535	51.3	6078	46.8	
Race					< 0.001
White	636	61.0	10,207	78.6	
Black	64	6.2	872	6.7	
Other	342	32.8	1912	14.7	
Histology					< 0.001
Adenocarcinoma	872	83.7	9802	75.5	
Other	170	16.3	3189	24.5	
Tumor size(cm)					< 0.001
≤ 4	566	54.3	8076	62.2	
> 4	476	45.7	4915	37.8	
Grade					< 0.001
Grade I	612	58.7	5759	44.3	
Grade II	274	26.3	3669	28.3	
Grade III	131	12.6	2628	20.2	
Grade IV	25	2.4	935	7.2	
FIGO					0.782
I	758	72.7	9312	71.7	
II	80	7.7	965	7.4	
III	155	14.9	2044	15.7	
IV	49	4.7	670	5.2	
Radiotherapy					< 0.001
Yes	239	22.9	3977	30.6	
No/Unknown	803	77.1	9014	69.4	
Chemotherapy					0.081
Yes	189	18.1	2650	20.4	
No/Unknown	853	81.7	10,341	79.6	
Surgery					0.128
Yes	1033	99.1	12,804	98.6	
No	9	0.9	187	1.4	

### Younger patient characteristics

The younger group comprised 101 (13.8%) patients ≤ 33 years old and 631 (86.2%) patients 33–44 years old. In the race group, 636(61.0%) patients were white, 64(6.2%) were black, and 342 (32.8%) patients were of other ethnic groups. Further, 507 (48.7%) were identified as married and 535 (51.3%) patients were in other marital statuses. Regarding the pathological type, adenocarcinoma was the most prevalent, accounting for 83.7% of all tumors. With respect to FIGO stage, the majority of patients were classified as FIGO I (72.7%). There were 566 (54.3%) patients with tumor size (cm) ≤ 4. The treatment protocols for the patients included chemotherapy (189; 18.1%) and radiotherapy (239; 22.9%). The proportion of patients undergoing surgery was 99.1%. (Table 2)

**Table 2:** Baseline demographic and clinicopathological characteristics with early-onset EC patients

Variables	The training cohort	The validation cohort	Total	cardinality	*P*
	No. (%)	No. (%)	No. (%)		
Total	732	310	1042		
Age (year)				0.001	0.98
≤ 33	101 (13.8)	43(13.9)	144(13.8)		
33–45	631(86.2)	267(86.1)	898(86.2)		
Marital status				0.09	0.77
Married	354(48.4)	153(49.4)	507(48.7)		
Other	378(51.6)	157(50.6)	535(51.3)		
Race				5.19	0.07
White	433(59.2)	203(65.5)	636(61.0)		
Black	43(5.9)	21(6.8)	64(6.2)		
Other	256(34.9)	86(27.7)	342(32.8)		
Histology				0.43	0.51
Adenocarcinoma	609(83.2)	263(84.8)	872(83.7)		
Other	123(16.8)	47(15.2)	170(16.3)		
Tumor size(cm)				0.58	0.45
≤ 4	392(53.6)	174(56.1)	566(54.3)		
> 4	340(46.4)	136(43.9)	476(45.7)		
Grade				0.43	0.93
Grade I	428(58.5)	184(59.4)	612(58.7)		
Grade II	191(26.1)	83(26.8)	274(26.3)		
Grade III	95(13.0)	36(11.6)	131(12.6)		
Grade IV	18(2.4)	7(2.2)	25(2.4)		
FIGO				0.49	0.92
I	528(72.1)	230(77.1)	758(72.7)		
II	57(7.8)	23(7.4)	80(7.7)		
III	111(15.2)	43(13.9)	154(14.8)		
IV	36(4.9)	14(4.5)	50(4.8)		
Radiotherapy				0.39	0.53
Yes	164(22.4)	75(24.2)	239(22.9)		
No/Unknown	568(77.6)	235(75.8)	803(77.1)		
Chemotherapy				2.09	0.15
Yes	141(19.3)	48(15.5)	189(18.1)		
No/Unknown	591(80.7)	262(84.5)	853(81.9)		
Surgery				1.00	0.67
Yes	725(99.0)	308(99.4)	1033(99.1)		
No	7(1.0)	2(0.6)	9(0.9)		

### Univariate and multivariate analysis

Univariate Cox analysis identified age, tumorsize, histology, grade, FIGO stage, radiation, chemotherapy, and surgery as risk factors significantly associated with overall survival. These risk factors were included in the multivariate analysis (*P*-value < 0.05). Age, grade, FIGO stage, surgery were independent risk factors for poor overall survival (Table 3).

**Table 3:** Univariate and multivariate analysis of variables related to OS in the training cohort. (n = 732)

Variables	Univariate analysis			Multivariate analysis		
	HR	95%Cl	*p-value*	HR	95%Cl	*p-value*
Age						
≤ 33	Ref			Ref		
34–45	2.81	1.14–6.94	0.025	4.047	1.59–10.28	0.003
Marital status						
Married	Ref					
Other	1.31	0.85–2.01	0.218			
Race						
White	Ref					
Black	1.84	0.87–3.92	0.113			
Other	1.39	0.89–2.17	0.150			
Histology						
Adenocarcinoma	Ref			Ref		
Other	1.84	1.13–2.99	0.014	0.93	0.55–1.56	0.773
Tumor size(cm)						
≤ 4	Ref			Ref		
> 4	2.16	1.39–3.36	0.001	1.68	1.00-2.81	0.049
Grade						
Grade I	Ref			Ref		
Grade II	2.36	1.29–4.32	0.005	1.71	0.89–3.29	0.105
Grade III	8.75	5.07–15.08	< 0.001	3.93	1.98–7.79	< 0.001
Grade IV	22.17	10.40-47.26	< 0.001	4.64	1.82–11.87	0.001
FIGO						
I	Ref			Ref		
II	1.63	0.63–4.20	0.316	1.21	0.44–3.35	0.714
III	4.86	2.86–8.25	< 0.001	3.24	1.47–6.30	0.003
IV	27.40	15.97–47.97	< 0.001	13.56	5.73–32.08	< 0.001
Radiotherapy						
Yes	Ref			Ref		
No/Unknown	0.50	0.31–0.77	0.002	1.69	1.00-2.85	0.051
Chemotherapy						
Yes	Ref			Ref		
No/Unknown	0.15	0.10–0.24	< 0.001	0.91	0.45–1.85	0.793
Surgery						
Yes						
No	24.22	9.66–60.73	< 0.001	13.16	4.54–38.18	< 0.001

### Nomogram Construction

**Fig. 3:**
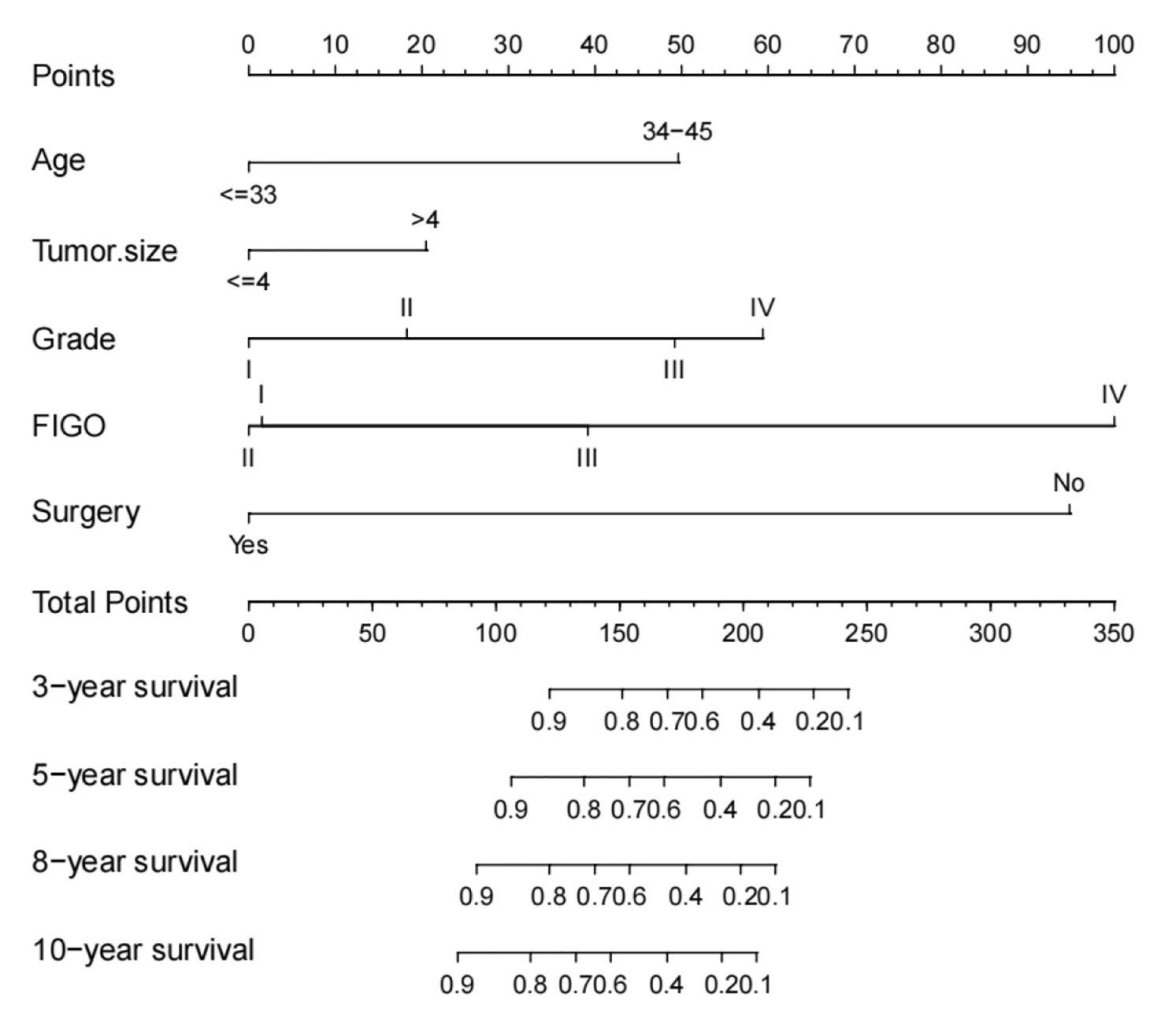
Nomogram for predicting 3-,5-, 8-and 10-year overall survival

The different subtypes of each independent prognostic variable were projected onto a scale to obtain a score for each item. The scores corresponding to independent prognostic factors were summed to obtain the total score. The higher the total score, the worse the prognosis. For a 40 year old EC patient with a tumorsize greater than 4 cm, grade II, FIGO stage II, and no surgical treatment, adding up the scores of each prognostic factor, she scored 183 points on the overall survival chart, and estimated a 5-year overall survival rate of 59% based on the nomogram. The nomogram showed that FIGO stage had the greatest influence on prognosis (Fig. 3).

### Internal validation

**Fig. 4:**
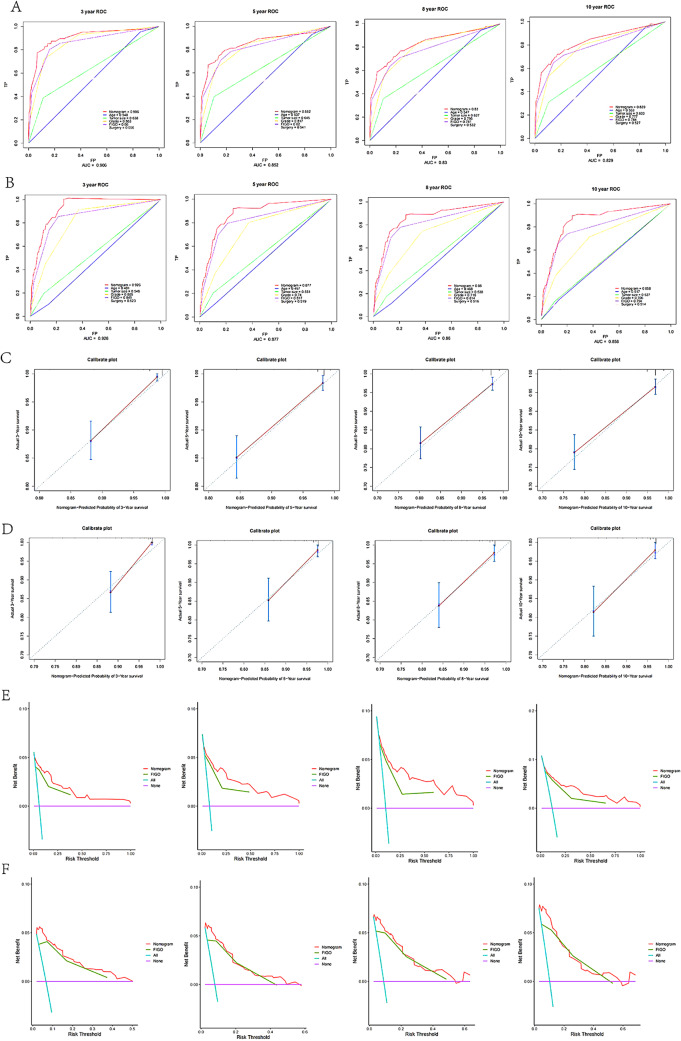
The area under the receiver operating characteristic curve (AUC value) was used to measure performance at 3-, 5-,8- and 10-year for the overall survival nomogram, age, tumorsize,grade, FIGO stag and surgery: (**A**) training cohort; (**B**) validation cohort; calibration curves for OS nomogram: (**C**) training cohort; (**D**) validation cohort; DCA curves for OS nomogram: (**E**) training cohort; (**F**) validation cohort

In the training cohort, the concordance index for the overall survival nomogram was 0.832(0.797–0.844), which was greater than that of a single independent prognostic risk factor. In the validation cohort, the concordance index for the nomogram of overall survival was 0.839 (0.810–0.868). This result was also superior to that of the independent prognostic risk factors. The AUC values for 3-, 5-, 8- and 10-year overall survival in the training cohort (0.926, 0.852, 0.830, and 0.829, respectively) were significantly higher than those for FIGO stage (0.849, 0.820, 0.781, and 0.784, respectively). The results for the validation cohort also showed significantly higher AUC values for the nomogram (0.926,0.877, 0.86, and 0.858) than for grade (0.849, 0.817, 0.814, and 0.794) (Fig. 4A, B). The calibration curves for the training and validation cohorts were close to the 45-degree diagonal, indicating that the probabilities of the predicted values were generally consistent with the actual probabilities (Fig. 4C, D). In addition, DCA curves confirmed the validity of the nomogram (Fig. 4E, F).

### External validation

In 70 early-onset EC patients, four were aged ≤ 33 years; among FIGO stage I were 84.3% (Supplementary Table 1). The AUCs at 5-, 8-, and 10-year for the nomogram (0.802, 0.807, and 0.791, respectively) were higher than those for the FIGO stage (0.713, 0.713, and 0.659) (Supplementary Fig. 1).

### Risk stratification

**Fig. 5:**
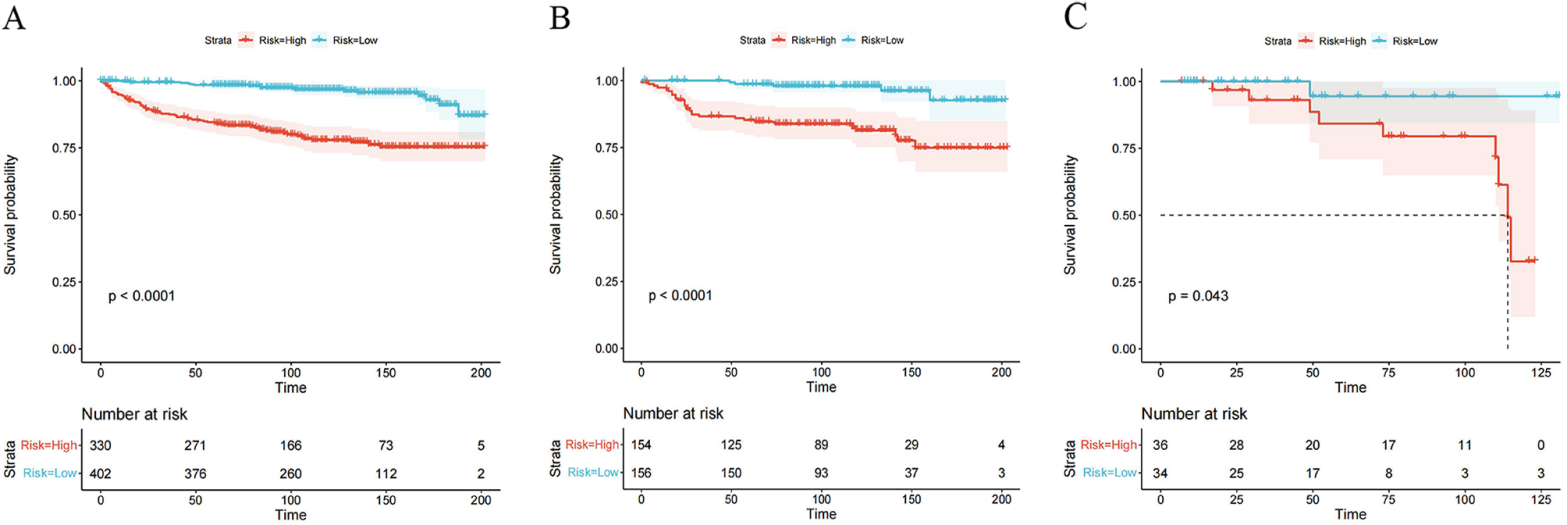
Kaplan-Meier survival curves for overall survival of early-onset EC patients in the high-risk and low-risk groups. (**A**) training cohort (score 51.12);(**B**) internal validation cohort (score 12.27); (**C**) external validation cohort

We divided the training cohort, the internal and external validation cohorts into high- and low-risk groups based on critical values. Comparison of overall survival between groups using Kaplan-Meier curves showed that overall survival rates were higher in all low-risk groups than in the high-risk group (*P*-value < 0.05) (Fig. 5).

## Discussion

The nomogram can transform complex regression equations into visualized simple graphs, making the prediction results easier to read and more convenient for evaluating patient conditions; In clinical practice, it can be used for multi-indicator joint diagnosis or prediction of disease risk or prognosis. By using nomogram, an accurate digital probability of survival or risk can be provided for each patient, which can assist clinical doctors in making decisions and reflect the idea of personalized healthcare.

We first compared the differences in the clinicopathological characteristics of the prognosis of younger and older patients with EC, and the results were consistent with previous studies [13, 14]. We further analyzed the clinicopathological characteristics of patients with EC aged < 45 years and examined the variables associated with their prognosis.

Race is widely recognized as an important risk factor for tumor prognosis [15, 16]. Tarney et al. [13] found that the median age at diagnosis of black patients with plasmacytoma and carcinosarcoma was 3 years younger than that of white patients (*P*-value < 0.0001). Some researchers have suggested that among EC patients, the mortality rate is 2.5 times higher in black women than in white women, despite a 30% lower incidence in black patients [16, 17]. This is primarily because patients respond differently to treatments, comorbidities, and genetic mutations. Our findings showed that white patients were more likely to develop EC, accounting for over 70% of the total. Research has found that histological type is an independent risk factor affecting prognosis [18]. As previously reported, adenocarcinoma is the predominant histological type of early onset EC [19]. The results of this study showed that the histological type was a prognostic factor for patients with early onset EC, but not an independent prognostic factor based on multifactorial analysis [20, 21]. Tumor grade also significantly affects patient prognosis, with survival rates decreasing as the tumor loses differentiation. By including 1,254 patients with stage I-II EC, Haley et al. showed that tumor grade and lymphatic vascular infiltration remained independent risk factors for prognosis in both younger and older patients [22]. As shown in the nomogram, tumor grade had a significant impact on prognosis in this study. Marriage is thought to be associated with a good prognosis in most cancers in women [23]. Unmarried women with cancer are at higher risk of late diagnosis and poorer survival outcomes than married women with cancer [24, 25]. The most frequent explanation is that marital status not only affects the regulation of the patient’s hormone levels but also has affects the patient’s psychosocial well-being. Lower et al. [26] 2015suggested that both marital status and relationship type are independent prognostic factors for survival in patients with EC. Our data showed that only half of the women were married. Tumor stage is a recognized prognostic factor for EC [27]. Higher staging suggests poorer prognosis. Liang et al. [28] reported that patients with high-grade EC were older than those with low-grade EC. A retrospective analysis by Pellerin et al. [20] found that 84.2% of EC patients under 45 years of age presented with stage I. However, by comparing the clinical characteristics and outcomes of patients with EC aged 45 years and younger with those aged 45 years and older, Evans-Metcalf et al. found the same overall distribution of tumor stage and survival in older patients compared with younger patients, a finding that contradicts several previous reports [29]. The staging system is a traditional tool used for assessing tumor prognosis. Most studies have shown that stage is an independent prognostic factor in patients with EC. TNM staging is the most widely accepted tumor staging system, and we used the 6th edition of the AJCC staging [30]. In this study, T-stage was an independent risk factor for overall survival in early-onset EC, and patients in T1 stage had a relatively good prognosis in 82.2% of cases. Tumor location is also an important factor in patient prognosis, with lymph node metastases or distant tumor metastases indicating a poor prognosis. Surgery is the main treatment method for early onset EC. Total Hysterectomy combined with bilateral tubal oophorectomy is the standard treatment, which can be performed by open or minimally invasive methods. The indications for adjuvant therapy are mainly based on clinical and pathological factors, such as age, grade, histology, depth of muscle infiltration, and lymphatic space infiltration [31]. Research has found that postoperative radiotherapy can significantly reduce the risk of local recurrence in women with moderate to high-risk EC [32].

Informatics is widely recognized as a tool for clinical research. Although the impact of different factors on EC prognosis has been studied, this has been limited to small-scale studies. To the best of our knowledge, this is the first study to use the SEER database to create a nomogram of overall survival in patients with early onset EC. However, the current study has some limitations: (I) Retrospective studies may lead to selection bias. (II) The SEER database is from the US, and a large amount of Chinese data is required for further validation. (III) The time period was 2004–2015, and we could only use the 6th edition of the AJCC.

Ultimately, our findings showed that younger patients had a better prognosis than older patients with a higher incidence of early- and low-grade disease. The standard treatment for patients with early onset EC is hysterectomy and bilateral salpingo-oophorectomy, with or without lymph node dissection. However, with socio-economic development and the implementation of China’s ‘two-child’ policy, EC management can be challenging for young women whose disease is mostly confined to the endometrium, with no extra-uterine metastases, and who have a strong desire to preserve their uterus [33–35]. The safety and efficacy of initial fertility-preserving treatment for patients with early stage, highly differentiated EC are now well established, and a more rigorous follow-up program must be adopted.

In summary, using the SEER database, we identified the factors associated with survival in patients with early onset EC, including age, marital status, race, histology, tumor size, grade, T stage, N stage, M stage, radiotherapy, chemotherapy and surgery. The development of the nomogram and use of a combination of internal and external validations demonstrated the good clinical applicability of the nomogram. A risk stratification system was created based on the risk scores generated by the nomogram. These findings may help clinicians tailor individual treatment plans for patients with early onset EC. However, further validation of our findings is required in prospective multicenter studies. With the development of precision medicine, traditional pathological classification can no longer meet the needs of clinical diagnosis and treatment. With the emergence of molecular classification, it will promote the combination of traditional pathological classification and molecular classification, in order to comprehensively evaluate the prognosis of endometrial cancer and effectively guide clinical treatment. As the main direction for further research in the future, the development of new fertility preserving treatment strategies based on molecular typing characteristics requires more and larger clinical trials.

### Electronic supplementary material

Below is the link to the electronic supplementary material.

Supplementary Material 1

Supplementary Material 2

## Data Availability

All data and materials supporting our fundings can be obtained from corresponding author upon request. Data were public for the sake of privacy and ethical restrictions. The original contributions presented in this study are included in the supplementary material, further inquiries can be directed to the corresponding authors. The website for obtaining data in this article was the SEER database (http://seer.cancer.gov/seerstat/). SEER*Stat 8.4.1.2. (account ID: 18893816203@163.com).

## Abbreviations

AJCC: American Joint Committee on Cancer (AJCC)
AUC: Area under the curve
CI: Confidence interval
SEER: Surveillance, epidemiology and end results
SPSS: Statistical product and service solutions
