# Supplementary material for: Development and validation of a nomogram for predicting overall survival in patients with early-onset endometrial cancer
Source: BMC Cancer. 2023 Dec 14;23:1230. doi: 10.1186/s12885-023-11682-9 (PMC10720131; doi:10.1186/s12885-023-11682-9)
Supplement: Supplementary file 1 — Supplementary Material 1 [file 12885_2023_11682_MOESM1_ESM.docx]

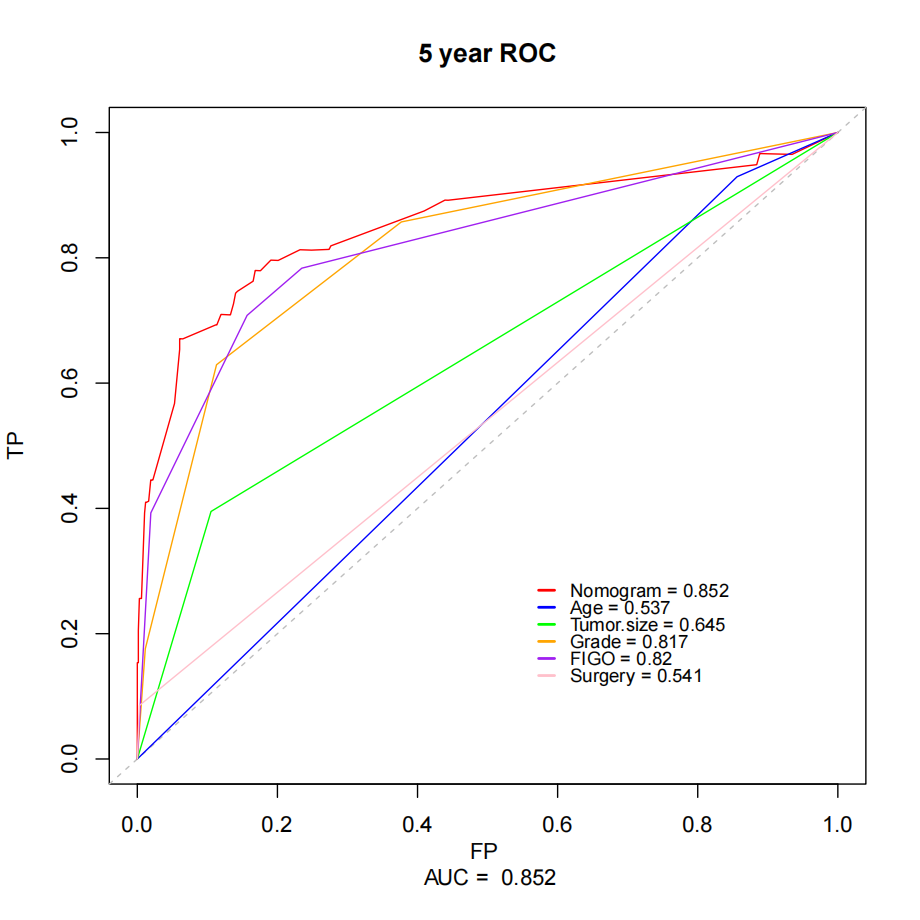

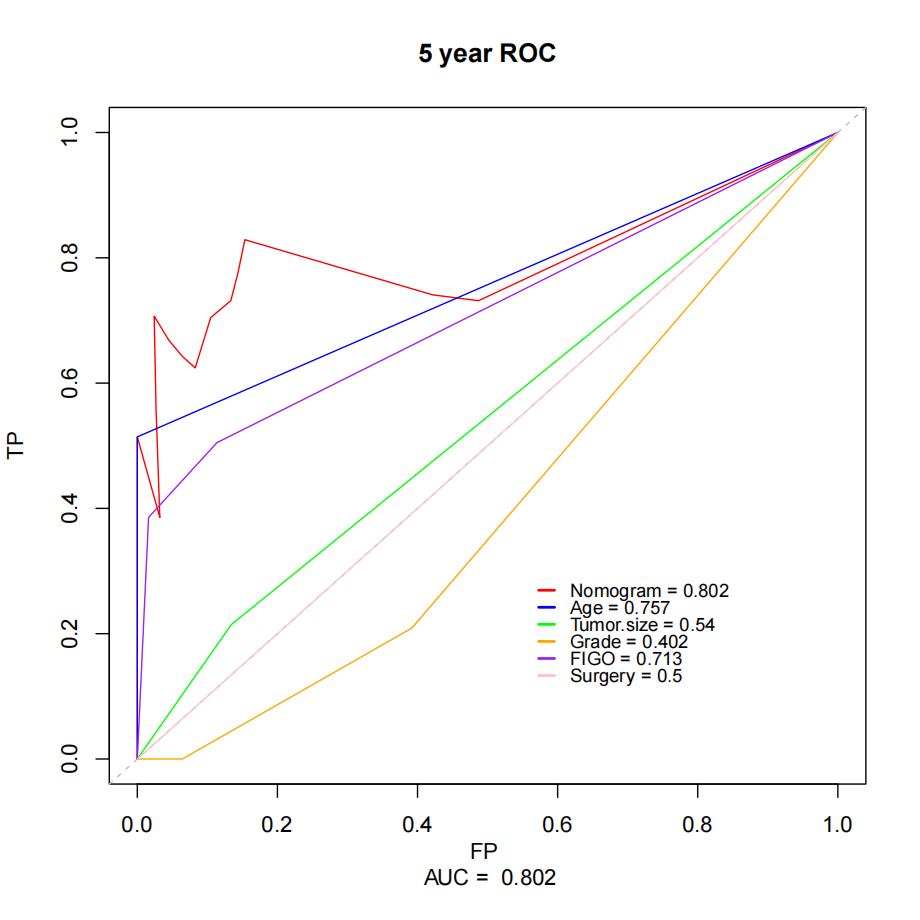

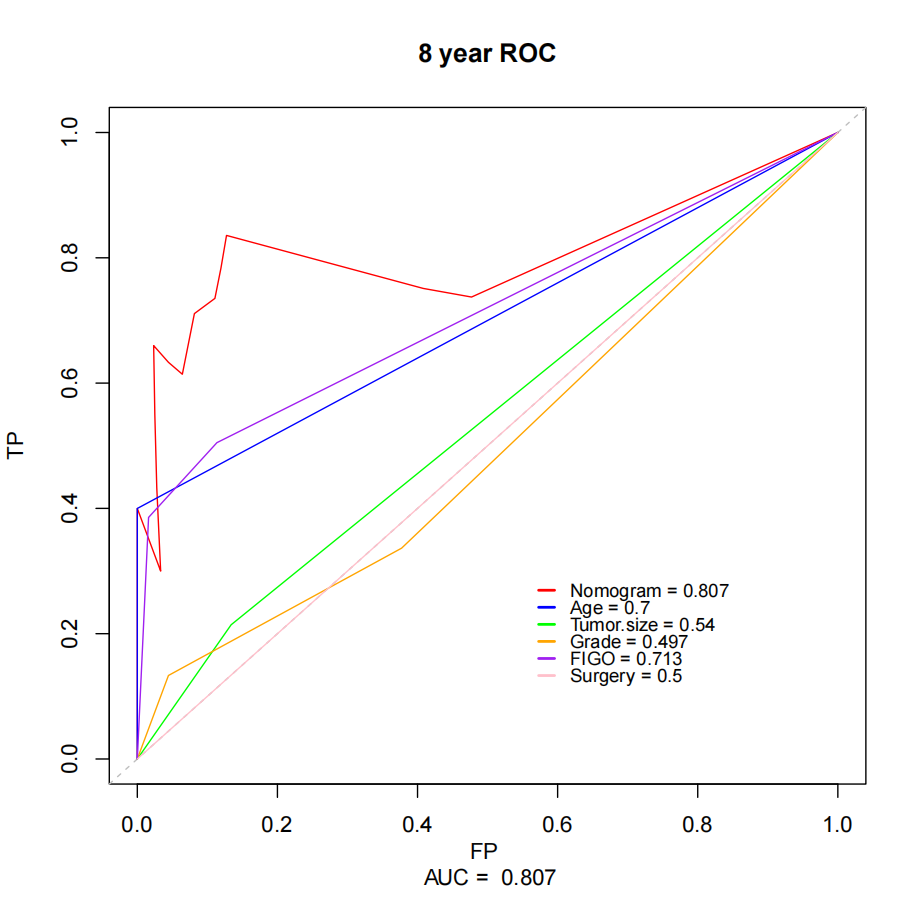


**Supplementary Fig. 1** ROC curves for the external validation cohort of 5-, 8- and 10-year (Nomogram, Age, Tumorsize,Grade,FIGO and Surgery).
