# Supplementary material for: Development and validation of a nomogram for predicting overall survival in patients with early-onset endometrial cancer
Source: BMC Cancer. 2023 Dec 14;23:1230. doi: 10.1186/s12885-023-11682-9 (PMC10720131; doi:10.1186/s12885-023-11682-9)
Supplement: Supplementary file 2 — Supplementary Material 2 [file 12885_2023_11682_MOESM2_ESM.docx]

**Supplementary Table 1** Clinical characteristics of patients with early-onset EC from the external validation cohort.

| Characteristics | Count | % |
| --- | --- | --- |
| Age |  |  |
| ≤ 33 | 4 | 5.7 |
| 33-44 | 66 | 94.3 |
| Tumor size(cm) |  |  |
| <4 | 60 | 85.7 |
| ≥ 4 | 4 | 5.7 |
| Grade |  |  |
| Grade I | 44 | 62.9 |
| Grade II | 22 | 31.4 |
| Grade III | 4 | 5.7 |
| Grade IV | 0 | 0 |
| FIGO stage |  |  |
| I | 59 | 84.3 |
| II | 7 | 10.0 |
| III | 4 | 5.7 |
| IV | 0 | 0 |
| Surgery |  |  |
| Yes | 70 | 100 |
| No | 0 | 0 |
